# Nursing Support for Pain in Patients With Cancer: A Scoping Review

**DOI:** 10.7759/cureus.49692

**Published:** 2023-11-30

**Authors:** Miharu Morikawa, Kohei Kajiwara, Masamitsu Kobayashi, Kanno Yusuke, Kimiko Nakano, Yoshinobu Matsuda, Yoichi Shimizu, Taichi Shimazu, Jun Kako

**Affiliations:** 1 Graduate School of Medicine, Kyoto University, Kyoto, JPN; 2 Faculty of Nursing, Japanese Red Cross Kyushu International College of Nursing, Munakata, JPN; 3 Graduate School of Nursing Science, St. Luke’s International University, Tokyo, JPN; 4 Department of Home Health and Palliative Care Nursing, Graduate School of Health Care Sciences, Tokyo Medical and Dental University, Tokyo, JPN; 5 Clinical Research Center for Developmental Therapeutics, Tokushima University Hospital, Tokushima, JPN; 6 Department of Psychosomatic Internal Medicine, National Hospital Organization Kinki-Chuo Chest Medical Center, Sakai, JPN; 7 Department of Adult Nursing, National College of Nursing, Japan, Tokyo, JPN; 8 Division of Behavioral Sciences, National Cancer Center Institute for Cancer Control, National Cancer Center, Tokyo, JPN; 9 Graduate School of Medicine, Mie University, Tsu, JPN

**Keywords:** end of life, scoping review, care, nursing, pain, cancer

## Abstract

Pain is subjective, warranting tailored responses in pharmacotherapy and nursing support. Despite this, the evidence for suitable nursing support for pain is not well established in terminally ill patients such as those with cancer; therefore, it is necessary to provide support in consideration of changes in physical symptoms and quality of life. However, interventional studies for such patients are often difficult. There have been no comprehensive studies to date on non-pharmacological support that can be implemented by nurses. Therefore, with the aim of examining nursing support applicable at the end of life, this scoping review comprehensively mapped nursing support for pain in cancer patients at all stages of the disease. This study complies with the Preferred Reporting Items for Systematic Reviews and Meta-Analyses (PRISMA) statement and the Arksey and O’Malley framework. All available published articles from the time of database establishment to January 31, 2022, were systematically searched for in PubMed, Cumulative Index to Nursing and Allied Health Literature (CINAHL), CENTRAL, and the Ichushi Web database of the Japanese Society of Medical Abstracts. Overall, 10,385 articles were screened, and 72 were finally included. Both randomized controlled trials (RCTs) (n = 62) and non-RCTs (n = 10) were included. Twenty-two types of nursing support were identified. Eighteen of them showed positive results; five of them were provided only to terminally ill patients, three of which were effective, namely, comfort care, foot bath, and combined therapy. It is important to examine the applicability of types of nursing support in clinical practice in the future.

## Introduction and background

Most cancer patients experience pain [1]; in particular, more than half of patients with advanced, metastatic, or terminal cancer experience pain [2]. In addition, cancer pain is a subjective experience and a complex symptom with varying factors (e.g., tumor or treatment-related and non-cancer-related), nature (e.g., nociceptive and neuropathic), and duration (e.g., persistent pain, sudden pain, and chronic pain), and its management requires an individualized approach. Therefore, in parallel with pharmacological therapy, nurses provide education on pain management and care to increase pain threshold through various activities such as foot bathing, positioning, and massage [3].

The National Comprehensive Cancer Network (NCCN) [4] and American Society of Clinical Oncology (ASCO) [5] guidelines for patients with pain at any stage of disease recommend a combination of pharmacological and non-pharmacological pain management strategies according to patient preferences. In terms of non-pharmacological therapy, the NCCN guidelines recommend physical interventions such as conditioning exercise, massage, heating and cooling, acupressure, and cognitive-behavioral interventions such as mindfulness, breathing techniques, and relaxation, as well as psychosocial support and spiritual care [4]. The ASCO guidelines recommend moderate acupuncture for joint pain due to the use of aromatase inhibitors and reflexology, massage, acupressure, yoga, and muscle relaxation therapy for general and musculoskeletal pain [5]. Only moderate massage is also recommended for patients with cancer receiving palliative care. However, evidence of suitable nursing support for specifically terminally ill patients is still insufficient.

In the case of terminally ill cancer patients with a prognosis of weeks until death, the increased distress of physical symptoms, decline in physical and cognitive functions, and psychological changes warrant special attention to the needs of the patient [6]. However, guidelines for nursing support for cancer pain and consensus on nursing support for terminally ill patients are lacking. The purpose of this scoping review was to map nursing support for pain in cancer patients at all phases of the disease before examining the potential of pain care for terminally ill cancer patients.

## Review

Objective and methodology

In this study, nursing support for pain is defined as any non-pharmacological treatment for pain relief that can be provided by a nurse. This study was conducted in accordance with a previously published protocol [6]. The protocol article states that information would be collected using the Delphi method to examine the feasibility of providing support to terminally ill cancer patients, although, in this study, we continued to map the results of the scoping review. We applied Arksey and O’Malley’s five-step scoping review framework [7] and followed the Preferred Reporting Items for Systematic Reviews and Meta-Analyses (PRISMA) extended version for scoping reviews (PRISMA-ScR) reporting guidelines [8].

Step 1: Identification of research questions

A systematic literature search was conducted on nursing support for cancer pain. The research question for this study was “What types of nursing support are provided to reduce cancer pain?”

Step 2: Identification of relevant research

PubMed, Cumulative Index to Nursing and Allied Health Literature (CINAHL), Cochrane Central Register of Controlled Trials in the Cochrane Library, and the Japanese Ichushi Web database of the Japanese Society of Medical Abstracts were searched from the time of database establishment to January 31, 2022. Relevant studies were evaluated from the list of articles, and major journals were manually searched. Search queries were first created in PubMed for an initial search, and then, search formulas were created to match other databases (refer to the protocol study) [6]. Two researchers (MM and JK) conducted this initial search in consultation with a librarian. Eligibility criteria were determined by physicians and nurses specializing in symptom management for patients with cancer. The inclusion criteria were studies that reported (a) patient age as 18 years or above, (b) interventions aimed at relieving pain, (c) nursing support, and (d) quantitative assessment of pain using a scale. The exclusion criteria were (a) studies that reported at least 20% non-cancer participants, (b) secondary analyses, and (c) those in languages other than Japanese and English. The details are described in the protocol article [6].

Step 3: Study selection process

**Figure 1:**
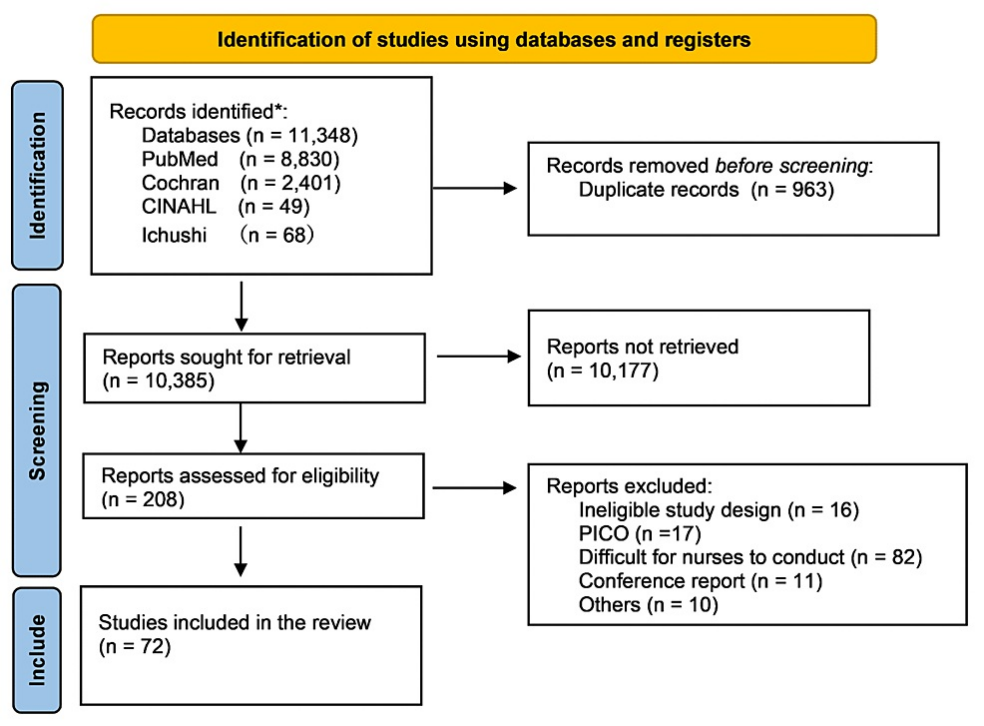
PRISMA flow diagram PRISMA: Preferred Reporting Items for Systematic Reviews and Meta-Analyses, PICO: population, intervention, comparison, and outcomes

Two investigators (MM and JK) independently evaluated the titles and abstracts of all studies and then screened the complete studies against the eligibility criteria. Discrepancies in study selection were resolved through discussion. The study selection process is summarized in Figure 1.

Step 4: Data charting

A form was developed to extract study characteristics, including the name of the first author, year of publication, country of publication, design of the study, size of the sample, eligible patients, type of nursing support, outcome measurement tools, and intervention effect. The same two researchers independently extracted the data. Studies that did not meet the eligibility criteria were excluded at this stage.

Step 5: Consolidation, summarization, and reporting of results

Nursing support reported in the articles extracted from the literature review was categorized by element of care using qualitative thematic analysis. First, the nursing support data described in the subject study were extracted into Microsoft Excel (Microsoft Corp., Redmond, WA) as raw data and analyzed for possible patterns, and notes were made as initial codes leading to the classification of the nursing support elements. Second, initial codes were created based on the intent and content of care provided. Third, patterns were searched, and potential nursing support components were grouped. Fourth, the initial codes were reviewed to find common nursing support components. Finally, each component was identified and clearly named. Several nursing support terms defined in the included studies were used as references. The analysis validated the classification by one author (MM) through discussion with another researcher (JK) and the entire study group.

Results

Our initial literature search listed 11,348 studies. After the removal of 963 duplicate studies, the titles and abstracts of 10,385 studies were screened, following which 10,177 studies were excluded. A total of 208 full-text studies were assessed for study eligibility and relevance, of which 72 were judged to satisfy the eligibility criteria (Figure 1). The interventions in the 72 studies were qualitatively categorized into 22 types of nursing support. Each intervention was divided according to the stage of the participant’s illness (all phases, treatment phase, terminal phase, and no notation); the number of tabulated studies is shown in Table 1. The details of the identified studies are presented in Table 2.

**Table 1:** Number of papers selected for secondary screening and classification of support

Intervention	Total	Participant’s phase of disease			
		All phases	Treatment phase	Terminal phase	No representation
Education programs focused on the provision of knowledge and information	10	7	2	0	1
Education focused on self-care management	11	10	0	0	1
Education using coaching skills	4	3	1	0	0
Education and psychological care	6	4	1	0	1
Exercise	2	1	1	0	0
Progressive muscle relaxation	2	1	1	0	0
Guided image therapy	3	2	0	0	1
Combination therapy (progressive muscle relaxation/guided image therapy/cognitive therapy)	4	3	0	1	0
Cognitive-behavioral intervention	4	2	2	0	0
Narrative approach	2	1	1	0	0
Relaxation therapy using virtual reality	2	2	0	0	0
Massage therapy	9	5	2	2	0
Aromatherapy massage	1	0	0	1	0
Reflexology	5	1	3	1	0
Music therapy	6	6	0	0	0
Poetry appreciation	1	1	0	0	0
Foot bath	1	0	0	1	0
Reiki	1	1	0	0	0
Self-administered acupressure	1	1	0	0	0
Auricular point acupressure	1	1	0	0	0
Comfort care (environmental adjustment/mental healthcare/oral care)	2	1	1	0	0
Adjustments to home care program	2	1	1	0	0

**Table 2:** Details of studies by nursing support type BPI^§^: Brief Pain Inventory, MPQ^†^: McGill Pain Questionnaire, NRS^€^: numerical rating scale, VAS^¶^: visual analog scale, EORTC-QLQ-C30^Θ^: European Organization for Research and Treatment of Cancer QLQ-C30, RCT^Δ^: randomized controlled trial, POS: pre- and post-test, N/A: not applicable/available, VR: virtual reality

Nursing support type	First author (publication year)	Country	Study design	Type of cancer	Phase of disease	Sample size (number)	Intervention	Outcome measure	Effect of pain reduction
Education programs focused on the provision of knowledge and information	Clotfelter (1999) [9]	USA	RCT^Δ^	Various	All	36	Pain management education using a booklet and a 14-minute video	VAS^¶^	〇
	Wells (2003) [10]	USA	RCT	Various	Treatment	310	Pain management education + home visits (2, 4, and 6 weeks) + telephonic follow-up (3 and 5 weeks)	BPI	〇
	Lai (2004) [11]	Turkey	RCT	Various	All	30	10-15 minutes × 5 days of pain management education using booklets	BPI	〇
	Anderson (2004) [12]	USA	RCT	Various	All	97	Pain management education using videos and booklets, answering questions, and telephonic follow-up	BPI	×
	Yildirim (2005) [13]	Canada	RCT	Various	All	39	5-15 minutes × 1-3 days of pain management education using slides and booklets	MPQ^†^, NRS^€^	〇
	Aubin (2006) [14]	USA	POS	Various	All	80	15-minute video followed by nurse answering questions, explanation using booklet, and recommendation of pain diary	BPI	〇
	Lovell (2010) [15]	Australia	RCT	Various	All	217	Pain management education using booklets, pain management education using videos, and pain management education using booklets and videos	BPI^§^	×
	Kim (2003) [16]	USA	RCT	Solid tumor	All	108	Pain management education using video and booklet + telephonic follow-up (after 1 week)	BPI	×
	Vallerand (2018) [17]	USA	RCT	Various	N/A	64	Pain management education + access to hotline, pain management education + telephonic follow-up	BPI	×
	Chee (2020) [18]	USA	RCT	Breast	Treatment	94	Online discussion, pain management education, and resource application	BPI	×
Education focused on self-care management	Kravitz (1996) [19]	USA	RCT	Various	All	78	Perform periodic pain assessments and record pain intensity levels on a bedside wall chart	VAS, MPQ	×
	de Wit (1997) [20]	Netherlands	RCT	Various	All	383	Pain education program (education on the basic principles of pain management, instruction on diary writing, and communication on pain) + telephonic follow-up at 2, 4, and 8 weeks	NRS	〇
	de Wit (1999) [21]	Netherlands	RCT	Various	All	159	Pain management program (information and education on pain management, explanation of how to describe it using a pain diary, communication with medical personnel) + telephonic follow-up on days 3 and 7 after discharge	EORTC-QLQ-C30^Θ^	〇
	Miaskowski (2004) [22]	Korea	RCT	Various	All	174	PRO-SELF Plus pain control program: face-to-face individual intervention (psychoeducational intervention and management of medications) phone calls (2, 4, and 5 weeks) + visits at 3 and 6 weeks	NRS	〇
	Miaskowski (2007) [23]	USA	RCT	Various	All	167	PRO-SELF Plus pain control program: (psychoeducational intervention, management of medications: how to manage medications and communicate with healthcare providers during the week using a medicine box), phone calls (2, 4, and 5 weeks) + visits at 3 and 6 weeks	BPI	×
	Koller (2013) [24]	Switzerland	RCT	Various	All	39	PRO-SELF Plus pain control program: face-to-face individual intervention (psychoeducational intervention, management + 6 visits and 4 telephonic follow-ups over a 10-week period of medications)	NRS	×
	Rustøen (2014) [25]	Norway	RCT	Various (with bone metastases)	All	179	PRO-SELF Plus pain control program: face-to-face individual intervention (booklet, pain diary, and pill box) + telephonic follow-up at 1, 3, and 6 weeks	EORTC-QLQ-C30	×
	Jahn (2014) [26]	Germany	RCT	Various	N/A	207	SCION-PAIN program (sessions to reduce misconceptions about pain and improve self-care skills) + telephonic follow-up within 3 days of discharge	BPI	×
	Koller (2018) [27]	Germany	RCT	Various	All	39	AntiPain program (information, skill building, and nurse coaching), booklets and pill boxes to organize oral medications + post-discharge telephonic follow-up	NRS	×
	Aliasgharpour (2018) [28]	Iran	RCT	Various	All	98	Self-management education (information, impact of pain on life, barriers to pain management, and introduction to pharmacological and non-pharmacological therapies): 20 minutes for each session	VAS	〇
	Musavi (2021) [29]	Iran	RCT	Various	All	82	Self-management education (information, acquisition of skills, and guidance) + telephonic follow-up	VAS	〇
Education using coaching skills	Yates (2004) [30]	Australia	RCT	Breast, colorectal, lung, or head and neck	All	189	Coaching interventions for knowledge and attitudes toward addressing patient-specific barriers to effective pain management, communication with healthcare providers, and reluctance to take analgesics × 2 sessions (30 minutes in person and 15 minutes by phone after 1 week)	NRS	×
	Wilkie (2010) [31]	USA	RCT	Lung	All	215	2 minutes of videotaped coaching to encourage self-monitoring + 5-10 minutes of in-person or phone coaching tailored to the patient’s pain	NRS	×
	Thomas (2012) [32]	USA	RCT	Various	All	289	Education group focusing on attitudes toward coping with barriers using videos and booklets and coaching group: 4 × 30-minute sessions using motivational interviewing techniques over the phone (beliefs about pain, pain management, and communication)	BPI	×
	Nguyen (2018) [33]	Vietnam	RCT	Various	Treatment	102	Provide information and education using iPad and PowerPoint + create a personalized symptom self-management plan based on Five A Model of Self-Management Support (identify patient concerns, barriers, etc.; set behavior change goals for them; and propose plans for improvement)	BPI	×
Education and psychological care	Ward (2000) [34]	USA	RCT	Gynecologic	All	43	Provide informational booklet + discussion of barriers to pain + telephonic follow-up after 1 week	BPI	〇
	Chang (2002) [35]	Taiwan	RCT	Various	N/A	37	Education using booklet: fatalism, addiction, desire to be good, fear of distracting physicians, disease progression, tolerance, side effects, and religious fatalism (30-40 minutes) + follow-up visit (2 weeks after discharge)	BPI	〇
	Ward (2009) [36]	Taiwan	RCT	Various	All	155	(1) Patient and significant others and (2) patient only: discuss beliefs about pain and concerns about analgesics and identify misconceptions and confusion + one (20-80-minute) session to fill in the gaps (2 or 4 weeks later, continue as needed)	BPI	(1)〇 (2)〇
	van der Meulen (2014) [37]	Netherlands	RCT	Head and neck	Treatment	205	Nurse-led counseling (advice, emotional support, education, and behavioral training) (45-60 minutes), beginning 6 weeks after cancer treatment ends, every other month over a 12-month period	EORTC-QLQ-C30	〇
	Kim (2018) [38]	Korea	RCT	Breast	All	60	A total of 7 weekly psychological intervention programs, counseling sessions in person or by telephone	EORTC-QLQ-C30	〇
	Zhang (2020) [39]	China	Non-RCT	Various	All	220	Patient education and psychological care provided after 30 minutes of regular medical care	NRS	×
Exercise	Rief (2014) [40]	Germany	RCT	Various	Treatment	81	Internet-based exercises (warm-up, cardio, and cool down), 90 minutes per session × 3 times per week for 8 weeks	VAS	〇
	Galiano-Castillo (2016) [41]	Spain	RCT	Breast	All	60	30 minutes of exercise to strengthen paraspinal muscles	BPI	〇
Progressive muscle relaxation	Kwekkeboom (2008) [42]	USA	RCT	Various	All	40	Muscle relaxation therapy with recorded sound sources: relax muscles in a series of 12 major muscle groups from head to feet (time: 13 minutes and 36 seconds)	NRS	〇
	Dikmen (2019) [43]	Turkey	RCT	Ovarian/cervical	Treatment	740	Muscle relaxation therapy sessions (20 minutes × 2 times/week × 8 weeks): toes, feet, legs, calves, butt, thighs, abdominal muscles, back muscles, chest, hands, biceps/triceps, shoulders, neck, face, and tongue	BPI	〇
Guided image therapy	Anderson (2006) [44]	USA	Non-RCT	Various	All	180	Listen to tapes on imagery-guided therapy (20 minutes × 5 times/week × 2 weeks)	BPI	×
	Kwekkeboom (2008) [42]	USA	RCT	Various	All	40	Scan and identify areas of pain and ask them to imagine replacing the pain with another sensation	NRS	〇
	Buyukbayram (2021) [45]	Turkey	RCT	Solid and hematologic	All	59	Lying in bed at 30-45° position: listening to instrumental music while viewing pictures of nature on the computer (15.53 minutes) × 3 consecutive days	VAS	〇
Cognitive-behavioral intervention	Anderson (2006) [44]	USA	RCT	Various	Treatment	86	Listen to and practice cognitive-behavioral therapy tapes (20 minutes × 5 times/week × 2 weeks)	BPI	〇
	Sikorskii (2009) [46]	USA	RCT	Various	All	59	Nurse-led symptom management, cognitive-behavioral therapy: 6 interventions over 8 weeks	NRS	×
	Kwekkeboom (2010) [47]	USA	POS	Various	All	30	12 cognitive-behavioral strategies (relaxation exercise, guided imagery, including recordings of nature sounds), listen as needed by the patient for 2 weeks	NRS	×
	Kwekkeboom (2012) [48]	USA	RCT	Various	Treatment	254	Cognitive-behavioral therapy (information, explanation, and overview of cognitive-behavioral therapy) (12 contents) + relaxation exercises: at least once a day × 2 weeks	NRS	×
Narrative approach	Cepeda (2008) [49]	USA	RCT	Various	All	198	Described the impact of cancer on life: once a week for 20 minutes or more for 3 weeks	NRS	×
	Crogan (2008) [50]	USA	RCT	Various	All	10	12 storytelling sessions: one 90-minute session (in a group facilitated by a nurse, telling and retelling a story focused on your illness and building community)	MPQ	×
Combination therapy (progressive muscle relaxation/guided image therapy/cognitive therapy)	Arathuzik (1994) [51]	USA	RCT	Breast	All	24	20 minutes of muscle relaxation therapy and 20 minutes of imagery visualization therapy for a total of 75 minutes and 20 minutes of muscle relaxation therapy and 20 minutes of imagery visualization therapy and use of a booklet on 23 ways to relieve pain for approximately 120 minutes or less	VAS	×
	Charalambous (2016) [52]	Cyprus	RCT	Breast/prostate	All	208	Breathing exercise sessions, followed by progressive muscular relaxation and finally, pleasure-guided imagery (to improve mood and physical well-being)	NRS, EORTC-QLQ-C30	〇
	De Paolis (2019) [53]	Italy	RCT	Various	Terminal	104	Progressive muscle relaxation, 20 minutes of imagery-guided therapy	ESAS-r, NRS	〇
	Chen (2021) [54]	China	RCT	Leukemia	All	30	Relaxation exercises (abdominal breathing, progressive muscle relaxation, and imagery induction) 30 minutes × 2 times/day × 4 weeks	Condensed Memorial Symptom Assessment Scale	〇
Relaxation therapy using virtual reality	Bani Mohammad (2019) [55]	Jordan	RCT	Breast	All	80	VR viewing	VAS	〇
	Ashley Verzwyvelt (2021) [56]	USA	RCT	Various	All	33	Wear VR glasses and headphones for 5-15 minutes while receiving chemotherapy and choose favorite video from 9 natural environments	MPQ	×
Massage therapy	Weinrich (1990) [57]	USA	RCT	Various	All	28	Swedish massage (back): 10 minutes	VAS	×
	Grealish (2000) [58]	Australia	POS	Various (with bone metastases)	All	87	Foot massage with non-scented oil: 10 minutes × 3 days (between 7 and 8 PM)	VAS	〇
	Smith (2002) [59]	USA	Non-RCT	Various	Treatment	41	Therapeutic massage: 15-30 minutes × 3 times/week	NRS	×
	Soden (2004) [60]	UK	RCT	Various	Terminal	42	Back massage using lavender aroma oil: 30 minutes per session × 1 session/week × 4 weeks	VAS	×
	Jane (2009) [61]	Taiwan	POS	Various	All	30	38-50-minute massage of highly innervated (head, hands, and feet) and less innervated (back and limbs) regions	VAS	〇
	Jane (2011) [62]	Taiwan	RCT	Various (with bone metastases)	All	72	38-50-minute massage of highly innervated (head, hands, and feet) and less innervated (back and limbs) regions	VAS	〇
	Wang (2015) [63]	Taiwan	RCT	Various (stage Ⅳ)	Terminal	80	Abdominal massage: 15 minutes × 2 times/day × 3 days	NRS	×
	Cutshall (2017) [64]	USA	POS	Various	All	40	Hand massage for 10-20 minutes before chemotherapy	VAS	×
	Uysal (2017) [65]	Turkey	RCT	Colorectal	Treatment	65	Classical foot massage: 10 minutes each side for a total of 20 minutes × 2 times/week × 5 weeks	EORTC-QLQ-C30	〇
Aromatherapy massage	Soden (2004) [60]	UK	RCT	Various	Terminal	30	Oil massage without fragrance: 30 minutes × 1 session/week × 4 weeks	VAS	×
Music therapy	Anderson (2006) [44]	USA	RCT	Various	All	59	Listening to music: 20 minutes × 5 times/week × 2 weeks	BPI	〇
	Huang (2010) [66]	Taiwan	RCT	Various	All	129	Music appreciation (choice of Taiwanese or American music, etc.): 30 minutes	VAS	〇
	Krishnaswamy (2016) [67]	India	Non-RCT	Various	All	14	Listening to music with headphones: 20 minutes	NRS	〇
	Arruda (2016) [68]	Brazil	RCT	Various	All	65	Listening to music (game music, animation, and other instrumental music, music with rhythm and regularity)	VAS	〇
	Bareh (2017) [69]	India	RCT	Various	All	50	Listen to music: 15-20 minutes × 2 times (morning and evening)/day × 5 days	NRS	〇
	Hsieh (2019) [70]	Taiwan	RCT	Breast	All	60	Listening to music (classical, pop music, and traditional music): 30 minutes × 5 times/week × every other week for 24 weeks	NRS	〇
Reflexology	Uysal (2017) [65]	Turkey	RCT	Colorectal	Treatment	65	Reflexology: 20 minutes for the right leg, 10 minutes for the left leg, total: 30 minutes × 2 times/week × 5 weeks	EORTC-QLQ-C30	〇
	Dikmen (2019) [43]	Turkey	RCT	Ovarian/cervical	Treatment	740	Reflexology: 2 × 30 minutes (during 16 visits in 8 weeks)	BPI	〇
	Mantoudi (2020) [71]	Greece	RCT	Various	Terminal	81	Reflexology: 30 minutes × 7 sessions	BPI	×
	Anderson (2021) [72]	USA	RCT	Various	All	40	Reflexology: 20-25 minutes	VAS	〇
	Göral Türkcü (2021) [73]	Turkey	RCT	Gynecological	Treatment	68	Reflexology: (2 days after chemotherapy), 30-45 minutes × 6 sessions	EORTC-QLQ-C30	〇
Poetry appreciation	Arruda (2016) [68]	Brazil	RCT	Various	All	65	Appreciation of poetry (select easy-to-understand poems about life, death, health, illness, love, happiness, pain, hope, etc.)	VAS	〇
Foot bath	Yamamoto (2011) [74]	Japan	RCT	Various	Terminal	18	Bed rest (20 minutes) → foot bath (30 minutes) → bed rest (20 minutes)	VAS	〇
Self-administered acupressure	Cheung (2020) [75]	China	RCT	Various	All	30	Self-administered acupressure (training: 2 hours × 2 times per week), follow-up: 1 hour once per week × 3 weeks + practice (2 times/day × 4 weeks)	BPI	×
Auricular point acupressure	Yeh (2016) [76]	USA	RCT	Breast	All	31	Application of auricular acupressure tape: apply for 5 days, changing once a week × 4 weeks	NRS	〇
Reiki	Buyukbayram (2021) [45]	Turkey	Non-RCT	Various	All	180	Reiki performed by placing the researcher’s hands 23 cm above the patient’s body (head, eyes, neck, chest, abdominal cavity, and inguinal and leg area): 25-30 minutes × 3 consecutive days (between 7 and 9 pm, avoiding sleep time)	VAS	〇
Comfort care (environmental adjustment/mental healthcare/oral care)	Ye (2021) [77]	China	RCT	Lung	All	86	Comfort nursing (environmental nursing: cleanliness, ventilation, visitor coordination, soundproofing; mental health nursing: understanding psychological status and needs, encouraging mood swings, etc.; skin and oral care: linen change every 2 days; oral mucosa care: mouth sores care - purification, pain management, aerosol inhalation to reduce expectoration for breathing problems, etc.)	NRS	〇
	Ma (2021) [78]	China	RCT	Advanced gastric	Terminal	136	Hospice care (active communication with the patient, playing music, education on preparing for death, changing positions, and adjusting diet)	VAS	〇
Adjustments to home care program	van der Peet (2009) [79]	Netherlands	RCT	Various	All	120	Home pain management/control program (booklet, pain diary, and contact for problems), 60-90-minute visits (weeks 1, 3, and 6)	BPI	〇
	Shi (2015) [80]	China	RCT	Nasopharyngeal	Treatment	180	Home pain management/control program: telephone follow-up 1 week after discharge + telephone call (2 weeks after that + home nursing care) (a specialist nurse develops and implements the home visit plan)	EORTC-QLQ-C30	〇

Of these studies, 62 were randomized controlled trials (RCTs), five were non-RCTs, and five were prospective observational studies. The United States accounted for the largest number of these studies (26 studies), followed by Taiwan (seven studies), China (six studies), Turkey (five studies), and other countries (28 studies). A total of six studies had been published in the 1990s, 25 in the 2000s, 36 in the 2010s, and 13 in the 2020s. In terms of pain assessment tools, the Brief Pain Inventory (BPI) was the most commonly used (24 studies), followed by a numerical rating scale (NRS) (23 studies), and the visual analog scale (VAS) (22 studies). These scales convert pain into a numerical value. Associated factors such as beliefs, barriers, and concerns about analgesics and pain management, which may have an influence on perception of pain, were not identified in this study.

The largest number of studies were related to education and were categorized into four types based on the characteristics of the educational programs. Studies focused on providing knowledge and information, self-care management, using coaching skills, and emphasizing education and psychological care. In total, 10 and 11 studies focused on providing knowledge and information [9-18] and self-care management [19-29], respectively, and four and six studies used coaching skills [30-33] and educational and psychological care interventions [34-39], respectively. A total of 31 studies on education of all types were extracted, 29 of which were RCTs, one a non-RCT, and one a prospective observational study. All studies included a treatment phase. Although only one study each for specific cancers such as that of the lung, breast, and head and neck was extracted, most of the studies involved patients with various cancer types.

For nursing support to promote physical activity, two cases each of exercise [40,41] and progressive muscle relaxation therapy [42,43] were identified. One study on exercise and muscle relaxation therapy was reported for all disease stages and one for the treatment stage, both of which were RCTs.

For nursing support of perception of pain, three guided imagery therapies [42,44,45] (two RCTs), four cognitive-behavioral interventions [44,46-48] (three RCTs), and two narrative approaches [49,50] (two RCTs) were identified. Four studies (all RCTs) of combined therapies [51-54] that considered both physical function and cognition were extracted. One study of combined therapy involved only terminally ill cancer patients [53].

The types of nursing support expected to promote comfort and relieve local tension included relaxation therapy using virtual reality (VR) in two studies [55,56] (one RCT), massage therapy in eight studies [57-65] (four RCTs), aromatherapy massage in one RCT [60], music therapy in six studies [44,66-70] (five RCTs), reflexology in five RCTs [43,65,71-73], poetry appreciation in one RCT [68], and foot bathing in one RCT [74].

Two studies on massage therapy [60,63] and one study each on aromatherapy massage therapy [60], reflexology [71], and foot bathing [74] were conducted only in the terminal phase, whereas the others were conducted in the treatment phase or all phases of the disease.

One study each of self-administered acupressure [75], auricular point acupressure [76], and Reiki [45] were identified as considering nursing support related to Eastern medicine. The studies of self-acupressure and auricular acupressure used RCT designs, whereas the study of Reiki used a non-RCT design. In addition, two comfort care (environmental adjustment/mental healthcare/oral care) [77,78] and two adjustments to home care programs [79,80] were identified, all of which were RCTs.

Of the 72 studies, 48 showed a statistically significant reduction in pain. These included 15 studies of education (five focused on the provision of knowledge and information, five on self-management, and five on education and psychological care), six of music therapy, four each of massage and reflexology, three of combination therapy, and two each of exercise, progressive muscle relaxation therapy, comfort care, and adjustments to home care programs. There was one study each of acupressure, poetry appreciation, cognitive-behavioral intervention, relaxation, foot bathing, and Reiki.

Discussion

This study is the first to comprehensively map nursing research on non-pharmacological therapies for cancer pain. We reviewed nursing support for patients from the treatment phase to the end of life and identified 72 types of nursing support. Only six studies were conducted exclusively on terminally ill patients.

Patient education was identified as a form of nursing support for cancer pain. Pain management education focused on providing knowledge and information, including the introduction of videos, slides, booklets, and online applications to the patients. Education focused on self-care comprised several support packages, including the PRO-SELF Plus pain control program (a pain education intervention program that frames Orem’s self-care theory), which provides information and pillboxes to correct misconceptions about opioids and enable effective medication and communication with healthcare providers. It has been reported that a patient’s ability to effectively self-manage cancer pain can be negatively affected by inadequate knowledge and negative attitudes. Therefore, it is expected that these interventions will be useful to ensure that patients have the correct knowledge and demonstrate appropriate self-care skills [81]. Oldenmenge et al. [82] reported that education of patients with cancer pain not only improved their knowledge about cancer pain but also alleviated pain in 31% of the studies. In their review, Koller et al. [83] divided the content of the educational intervention into four components: cognitive, behavioral, goal setting, and direct contact between research staff and clinicians. They reported that interventions could not be clearly categorized by the educational component; although the present study also focused on the characteristics of educational interventions and categorized them, the components were not clearly separated. Nevertheless, as the purpose of the current study was to comprehensively map nursing support, the objective was achieved. Many of the studies identified in this study were educational for various cancer types and all disease phases, suggesting that the support is adaptable to many cancer patients. In contrast, many studies reported long-term interventions that included follow-up and should be scrutinized when considering their application to terminally ill patients.

Exercise was identified as an aid in promoting physical activity. Meta-analyses on exercise therapy for cancer pain management have shown that it is effective in reducing pain, although the effect size is small [84]. It has been suggested that exercise be tailored to the patient, as generalized exercise may be ineffective or lead to worsening of pain depending on the patient’s situation [84,85]. Similarly, muscle relaxation therapy has been shown to be effective, but the level of evidence is low [86], and evidence building is important before considering it as nursing support. The muscle relaxation and guided imagery therapies extracted in this study have been examined using recorded data, and such interventions would be easier for nurses to introduce in clinical practice. In this study, nursing support that can provide comfort and relieve local tension included relaxation using VR, combination therapy (progressive muscle relaxation/guided imagery therapy/cognitive therapy), massage, reflexology, foot bathing, music therapy, and poetry appreciation. Notably, many types of support were identified that address the diverse needs of patients and play a complementary role in pain management. Although a systematic review conducted on the effects of massage on cancer patient symptoms suggests that it has a beneficial effect on pain [87], only two of the six studies reported pain relief. This may partly be attributed to the fact that this study excluded treatments performed by qualified therapists and included those that could be performed by nurses and other medical personnel. It should also be noted that while differences in techniques used between practitioners are generally noted [88], it is more difficult to standardize techniques when they are performed by nurses than therapists. More effective comfort care interventions to raise patient pain threshold [3] should be studied, and relevant evidence should be built in the future.

Reiki, auricular acupressure, and self-acupressure instructions were also identified as other types of nursing support based on Eastern medicine, which are also referred to in the ASCO guidelines [5] as non-pharmacological therapies. In this study, these nursing support items were extracted in a format that nurses could easily incorporate into their clinical practice, such as auricular point acupressure, in which seeds are applied to the ear, and self-administered acupressure. Further study is needed to determine whether these can be implemented in clinical practice and the effectiveness of their implementation by nurses.

In addition, it was suggested that preparing the patient’s living and recuperation environment, including adjustments to comfort care and home care programs, is a fundamental element of nursing, and these are also important types of support in cancer pain management [77-80].

This scoping review has some limitations. First, because the search was limited to studies published in Japanese and English, this review may have excluded relevant studies published in other languages. Second, this scoping review was not designed to assess methodological quality, as its purpose was to map nursing support. Thus, this conclusion is primarily based on the extraction of nursing support investigated in studies rather than on the effectiveness of nursing support for cancer pain.

## Conclusions

In this study, we comprehensively mapped the non-pharmacological support provided by nurses for cancer pain and identified 22 types of nursing support from 72 studies. Of these, six studies were exclusively of terminally ill cancer patients, and only five types of nursing support were identified. The most common nursing support for cancer pain was related to education. Other types of support included those pertaining to the patient’s perception of pain, promoting patient comfort (believed to raise pain threshold), and adjusting the patient’s care environment. Further research on and consideration of the possible support for terminally ill patients are needed in the future.

## Appendices

Table 3 presents the PRISMA-ScR checklist.

**Table 3:** PRISMA-ScR checklist JBI: Joanna Briggs Institute, PRISMA-ScR: Preferred Reporting Items for Systematic Reviews and Meta-Analyses extension for Scoping Reviews *Where sources of evidence (see second footnote) are compiled from, such as bibliographic databases, social media platforms, and Web sites. †A more inclusive/heterogeneous term used to account for the different types of evidence or data sources (e.g., quantitative and/or qualitative research, expert opinion, and policy documents) that may be eligible in a scoping review as opposed to only studies. This is not to be confused with information sources (see first footnote). ‡The framework by Arksey and O’Malley (6) and Levac and colleagues (7) and the JBI guidance (4 and 5) refer to the process of data extraction in a scoping review as data charting. §The process of systematically examining research evidence to assess its validity, results, and relevance before using it to inform a decision. This term is used for items 12 and 19 instead of “risk of bias” (which is more applicable to systematic reviews of interventions) to include and acknowledge the various sources of evidence that may be used in a scoping review (e.g., quantitative and/or qualitative research, expert opinion, and policy document).

Section	Item	PRISMA-ScR checklist item	Reported on page #
Title			
Title	1	Identify the report as a scoping review.	P1
Abstract			
Structured summary	2	Provide a structured summary that includes (as applicable): background, objectives, eligibility criteria, sources of evidence, charting methods, results, and conclusions that relate to the review questions and objectives.	P3-4
Introduction			
Rationale	3	Describe the rationale for the review in the context of what is already known and explain why the review questions/objectives lend themselves to a scoping review approach.	P6
Objectives	4	Provide an explicit statement of the questions and objectives being addressed with reference to their key elements (e.g., population or participants, concepts, and context) or other relevant key elements used to conceptualize the review questions and/or objectives.	P6
Methods			
Protocol and registration	5	Indicate whether a review protocol exists; state if and where it can be accessed (e.g., a Web address); and if available, provide registration information, including the registration number.	P6-7
Eligibility criteria	6	Specify characteristics of the sources of evidence used as eligibility criteria (e.g., years considered, language, and publication status), and provide a rationale.	P8
Information sources*	7	Describe all information sources in the search (e.g., databases with dates of coverage and contact with authors to identify additional sources), as well as the date the most recent search was executed.	P7
Search	8	Present the full electronic search strategy for at least 1 database, including any limits used, such that it could be repeated.	P6 (#6; protocol paper)
Selection of sources of evidence†	9	State the process for selecting sources of evidence (i.e., screening and eligibility) included in the scoping review.	P7-8
Data charting process‡	10	Describe the methods of charting data from the included sources of evidence (e.g., calibrated forms or forms that have been tested by the team before their use, and whether data charting was done independently or in duplicate) and any processes for obtaining and confirming data from investigators.	P8-9
Data items	11	List and define all variables for which data were sought and any assumptions and simplifications made.	P8-9
Critical appraisal of individual sources of evidence§	12	If done, provide a rationale for conducting a critical appraisal of included sources of evidence and describe the methods used and how this information was used in any data synthesis (if appropriate).	-
Synthesis of results	13	Describe the methods of handling and summarizing the data that were charted.	P8-9
Results			
Selection of sources of evidence	14	Give numbers of sources of evidence screened, assessed for eligibility, and included in the review, with reasons for exclusions at each stage, ideally using a flow diagram.	Figure 1, P28
Characteristics of sources of evidence	15	For each source of evidence, present characteristics for which data were charted and provide the citations.	Tables 1 and 2, P29-42
Critical appraisal within sources of evidence	16	If done, present data on critical appraisal of included sources of evidence (see item 12).	-
Results of individual sources of evidence	17	For each included source of evidence, present the relevant data that were charted that relate to the review questions and objectives.	Tables 1 and 2, P29-42
Synthesis of results	18	Summarize and/or present the charting results as they relate to the review questions and objectives.	Tables 1 and 2, P29-42
Discussion			
Summary of evidence	19	Summarize the main results (including an overview of concepts, themes, and types of evidence available), link to the review questions and objectives, and consider the relevance to key groups.	P13-17
Limitations	20	Discuss the limitations of the scoping review process.	P16-17
Conclusions	21	Provide a general interpretation of the results with respect to the review questions and objectives, as well as potential implications and/or next steps.	P17
Funding			
Funding	22	Describe sources of funding for the included sources of evidence, as well as sources of funding for the scoping review and describe the role of the funders of the scoping review.	P18-19

## References

[1] Sheinfeld Gorin S, Krebs P, Badr H (2012). Meta-analysis of psychosocial interventions to reduce pain in patients with cancer. J Clin Oncol.

[2] Snijders RA, Brom L, Theunissen M, van den Beuken-van Everdingen MH (2023). Update on prevalence of pain in patients with cancer 2022: a systematic literature review and meta-analysis. Cancers (Basel).

[3] Twycross RG (1984). Control of pain. J R Coll Physicians Lond.

[4] Swarm RA, Paice JA, Anghelescu DL (2019). Adult cancer pain, version 3.2019, NCCN Clinical Practice Guidelines in Oncology. J Natl Compr Canc Netw.

[5] Mao JJ, Ismaila N, Bao T (2022). Integrative medicine for pain management in oncology: Society for Integrative Oncology-ASCO guideline. J Clin Oncol.

[6] Kako J, Kobayashi M, Kanno Y (2022). Nursing support for symptoms in patients with cancer and caregiver burdens: a scoping review protocol. BMJ Open.

[7] Arksey H, O’Malley L (2005). Scoping studies: towards a methodological framework. Int J Soc Res Methodol Theory Prac.

[8] Tricco AC, Lillie E, Zarin W (2018). PRISMA extension for scoping reviews (PRISMA-ScR): checklist and explanation. Ann Intern Med.

[9] Clotfelter CE (1999). The effect of an educational intervention on decreasing pain intensity in elderly people with cancer. Oncol Nurs Forum.

[10] Wells N, Hepworth JT, Murphy BA, Wujcik D, Johnson R (2003). Improving cancer pain management through patient and family education. J Pain Symptom Manage.

[11] Lai YH, Guo SL, Keefe FJ, Tsai SL, Chien CC, Sung YC, Chen ML (2004). Effects of brief pain education on hospitalized cancer patients with moderate to severe pain. Support Care Cancer.

[12] Anderson KO, Mendoza TR, Payne R (2004). Pain education for underserved minority cancer patients: a randomized controlled trial. J Clin Oncol.

[13] Yildirim YK, Cicek F, Uyar M (2009). Effects of pain education program on pain intensity, pain treatment satisfaction, and barriers in Turkish cancer patients. Pain Manag Nurs.

[14] Aubin M, Vézina L, Parent R (2006). Impact of an educational program on pain management in patients with cancer living at home. Oncol Nurs Forum.

[15] Lovell MR, Forder PM, Stockler MR (2010). A randomized controlled trial of a standardized educational intervention for patients with cancer pain. J Pain Symptom Manage.

[16] Kim HS, Shin SJ, Kim SC (2013). Randomized controlled trial of standardized education and telemonitoring for pain in outpatients with advanced solid tumors. Support Care Cancer.

[17] Hazard Vallerand A, Hasenau SM, Robinson-Lane SG, Templin TN (2018). Improving functional status in African Americans with cancer pain: a randomized clinical trial. Oncol Nurs Forum.

[18] Chee W, Lee Y, Ji X, Chee E, Im EO (2020). The preliminary efficacy of a technology-based cancer pain management program among Asian American breast cancer survivors. Comput Inform Nurs.

[19] Kravitz RL, Delafield JP, Hays RD, Drazin R, Conolly M (1996). Bedside charting of pain levels in hospitalized patients with cancer: a randomized controlled trial. J Pain Symptom Manage.

[20] de Wit R, van Dam F, Zandbelt L (1997). A pain education program for chronic cancer pain patients: follow-up results from a randomized controlled trial. Pain.

[21] de Wit R, van Dam F, Hanneman M (1999). Evaluation of the use of a pain diary in chronic cancer pain patients at home. Pain.

[22] Miaskowski C, Dodd M, West C, Schumacher K, Paul SM, Tripathy D, Koo P (2004). Randomized clinical trial of the effectiveness of a self-care intervention to improve cancer pain management. J Clin Oncol.

[23] Miaskowski C, Dodd M, West C, Paul SM, Schumacher K, Tripathy D, Koo P (2007). The use of a responder analysis to identify differences in patient outcomes following a self-care intervention to improve cancer pain management. Pain.

[24] Koller A, Miaskowski C, De Geest S, Opitz O, Spichiger E (2013). Results of a randomized controlled pilot study of a self-management intervention for cancer pain. Eur J Oncol Nurs.

[25] Rustøen T, Valeberg BT, Kolstad E, Wist E, Paul S, Miaskowski C (2014). A randomized clinical trial of the efficacy of a self-care intervention to improve cancer pain management. Cancer Nurs.

[26] Jahn P, Kuss O, Schmidt H (2014). Improvement of pain-related self-management for cancer patients through a modular transitional nursing intervention: a cluster-randomized multicenter trial. Pain.

[27] Koller A, Gaertner J, De Geest S, Hasemann M, Becker G (2018). Testing the implementation of a pain self-management support intervention for oncology patients in clinical practice: a randomized controlled pilot study (ANtiPain). Cancer Nurs.

[28] Aliasgharpour M, Davodabady F, Sajadi M, Razi SP, Kazem-Nejad A (2019). The effect of pain management training on the severity of pain in patients with cancer: a clinical trial study. Iran Red Crescent Med J.

[29] Musavi M, Jahani S, Asadizaker M, Maraghi E, Razmjoo S (2021). The effect of pain self-management education on pain severity and quality of life in metastatic cancer patients. Asia Pac J Oncol Nurs.

[30] Yates P, Edwards H, Nash R (2004). A randomized controlled trial of a nurse-administered educational intervention for improving cancer pain management in ambulatory settings. Patient Educ Couns.

[31] Wilkie D, Berry D, Cain K (2010). Effects of coaching patients with lung cancer to report cancer pain. West J Nurs Res.

[32] Thomas ML, Elliott JE, Rao SM, Fahey KF, Paul SM, Miaskowski C (2012). A randomized, clinical trial of education or motivational-interviewing-based coaching compared to usual care to improve cancer pain management. Oncol Nurs Forum.

[33] Nguyen LT, Alexander K, Yates P (2018). Psychoeducational intervention for symptom management of fatigue, pain, and sleep disturbance cluster among cancer patients: a pilot quasi-experimental study. J Pain Symptom Manage.

[34] Ward S, Donovan HS, Owen B, Grosen E, Serlin R (2000). An individualized intervention to overcome patient-related barriers to pain management in women with gynecologic cancers. Res Nurs Health.

[35] Chang MC, Chang YC, Chiou JF, Tsou TS, Lin CC (2002). Overcoming patient-related barriers to cancer pain management for home care patients. A pilot study. Cancer Nurs.

[36] Ward SE, Serlin RC, Donovan HS, Ameringer SW, Hughes S, Pe-Romashko K, Wang KK (2009). A randomized trial of a representational intervention for cancer pain: does targeting the dyad make a difference?. Health Psychol.

[37] van der Meulen IC, May AM, de Leeuw JR, Koole R, Oosterom M, Hordijk GJ, Ros WJ (2014). Long-term effect of a nurse-led psychosocial intervention on health-related quality of life in patients with head and neck cancer: a randomised controlled trial. Br J Cancer.

[38] Kim YH, Choi KS, Han K, Kim HW (2018). A psychological intervention programme for patients with breast cancer under chemotherapy and at a high risk of depression: a randomised clinical trial. J Clin Nurs.

[39] Zhang C, Guo H, Shen M, Chen G (2020). Comparison of clinical effectiveness of nurse led care among Chinese patients with cancer: a prospective study evaluating effective patient care compared to consultant oncologist. J Infect Public Health.

[40] Rief H, Omlor G, Akbar M (2014). Feasibility of isometric spinal muscle training in patients with bone metastases under radiation therapy - first results of a randomized pilot trial. BMC Cancer.

[41] Galiano-Castillo N, Cantarero-Villanueva I, Fernández-Lao C, Ariza-García A, Díaz-Rodríguez L, Del-Moral-Ávila R, Arroyo-Morales M (2016). Telehealth system: a randomized controlled trial evaluating the impact of an internet-based exercise intervention on quality of life, pain, muscle strength, and fatigue in breast cancer survivors. Cancer.

[42] Kwekkeboom KL, Wanta B, Bumpus M (2008). Individual difference variables and the effects of progressive muscle relaxation and analgesic imagery interventions on cancer pain. J Pain Symptom Manage.

[43] Dikmen HA, Terzioglu F (2019). Effects of reflexology and progressive muscle relaxation on pain, fatigue, and quality of life during chemotherapy in gynecologic cancer patients. Pain Manag Nurs.

[44] Anderson KO, Cohen MZ, Mendoza TR, Guo H, Harle MT, Cleeland CS (2006). Brief cognitive-behavioral audiotape interventions for cancer-related pain: immediate but not long-term effectiveness. Cancer.

[45] Buyukbayram Z, Citlik Saritas S (2021). The effect of Reiki and guided imagery intervention on pain and fatigue in oncology patients: a non-randomized controlled study. Explore (NY).

[46] Sikorskii A, Given CW, You M, Jeon S, Given BA (2009). Response analysis for multiple symptoms revealed differences between arms of a symptom management trial. J Clin Epidemiol.

[47] Kwekkeboom KL, Abbott-Anderson K, Wanta B (2010). Feasibility of a patient-controlled cognitive-behavioral intervention for pain, fatigue, and sleep disturbance in cancer. Oncol Nurs Forum.

[48] Kwekkeboom KL, Abbott-Anderson K, Cherwin C, Roiland R, Serlin RC, Ward SE (2012). Pilot randomized controlled trial of a patient-controlled cognitive-behavioral intervention for the pain, fatigue, and sleep disturbance symptom cluster in cancer. J Pain Symptom Manage.

[49] Cepeda MS, Chapman CR, Miranda N (2008). Emotional disclosure through patient narrative may improve pain and well-being: results of a randomized controlled trial in patients with cancer pain. J Pain Symptom Manage.

[50] Crogan NL, Evans BC, Bendel R (2008). Storytelling intervention for patients with cancer: part 2--pilot testing. Oncol Nurs Forum.

[51] Arathuzik D (1994). Effects of cognitive-behavioral strategies on pain in cancer patients. Cancer Nurs.

[52] Charalambous A, Giannakopoulou M, Bozas E, Marcou Y, Kitsios P, Paikousis L (2016). Guided imagery and progressive muscle relaxation as a cluster of symptoms management intervention in patients receiving chemotherapy: a randomized control trial. PLoS One.

[53] De Paolis G, Naccarato A, Cibelli F (2019). The effectiveness of progressive muscle relaxation and interactive guided imagery as a pain-reducing intervention in advanced cancer patients: a multicentre randomised controlled non-pharmacological trial. Complement Ther Clin Pract.

[54] Chen F, Mao L, Wang Y, Xu J, Li J, Zheng Y (2021). The feasibility and efficacy of self-help relaxation exercise in symptom distress in patients with adult acute leukemia: a pilot randomized controlled trial. Pain Manag Nurs.

[55] Bani Mohammad E, Ahmad M (2019). Virtual reality as a distraction technique for pain and anxiety among patients with breast cancer: a randomized control trial. Palliat Support Care.

[56] Ashley Verzwyvelt L, McNamara A, Xu X, Stubbins R (2021). Effects of virtual reality v. biophilic environments on pain and distress in oncology patients: a case-crossover pilot study. Sci Rep.

[57] Weinrich SP, Weinrich MC (1990). The effect of massage on pain in cancer patients. Appl Nurs Res.

[58] Grealish L, Lomasney A, Whiteman B (2000). Foot massage. A nursing intervention to modify the distressing symptoms of pain and nausea in patients hospitalized with cancer. Cancer Nurs.

[59] Smith MC, Kemp J, Hemphill L, Vojir CP (2002). Outcomes of therapeutic massage for hospitalized cancer patients. J Nurs Scholarsh.

[60] Soden K, Vincent K, Craske S, Lucas C, Ashley S (2004). A randomized controlled trial of aromatherapy massage in a hospice setting. Palliat Med.

[61] Jane SW, Wilkie DJ, Gallucci BB, Beaton RD, Huang HY (2009). Effects of a full-body massage on pain intensity, anxiety, and physiological relaxation in Taiwanese patients with metastatic bone pain: a pilot study. J Pain Symptom Manage.

[62] Jane SW, Chen SL, Wilkie DJ (2011). Effects of massage on pain, mood status, relaxation, and sleep in Taiwanese patients with metastatic bone pain: a randomized clinical trial. Pain.

[63] Wang TJ, Wang HM, Yang TS, Jane SW, Huang TH, Wang CH, Lin YH (2015). The effect of abdominal massage in reducing malignant ascites symptoms. Res Nurs Health.

[64] Cutshall SM, Mahapatra S, Hynes RS (2017). Hand massage for cancer patients undergoing chemotherapy as outpatients: a pilot study. Explore (NY).

[65] Uysal N, Kutlutürkan S, Uğur I (2017). Effects of foot massage applied in two different methods on symptom control in colorectal cancer patients: randomised control trial. Int J Nurs Pract.

[66] Huang ST, Good M, Zauszniewski JA (2010). The effectiveness of music in relieving pain in cancer patients: a randomized controlled trial. Int J Nurs Stud.

[67] Krishnaswamy P, Nair S (2016). Effect of music therapy on pain and anxiety levels of cancer patients: a pilot study. Indian J Palliat Care.

[68] Arruda MA, Garcia MA, Garcia JB (2016). Evaluation of the effects of music and poetry in oncologic pain relief: a randomized clinical trial. J Palliat Med.

[69] Bareh S, D’silva F (2017). Effect of music therapy on pain and quality of life among cancer survivors. J Health Allied Sci NU.

[70] Hsieh FC, Miao NF, Tseng IJ (2019). Effect of home-based music intervention versus ambient music on breast cancer survivors in the community: a feasibility study in Taiwan. Eur J Cancer Care (Engl).

[71] Mantoudi A, Parpa E, Tsilika E (2020). Complementary therapies for patients with cancer: reflexology and relaxation in Integrative palliative care. A randomized controlled comparative study. J Altern Complement Med.

[72] Anderson KD, Downey M (2021). Foot reflexology: an intervention for pain and nausea among inpatients with cancer. Clin J Oncol Nurs.

[73] Göral Türkcü S, Özkan S (2021). The effects of reflexology on anxiety, depression and quality of life in patients with gynecological cancers with reference to Watson's theory of human caring. Complement Ther Clin Pract.

[74] Yamamoto K, Nagata S (2011). Physiological and psychological evaluation of the wrapped warm footbath as a complementary nursing therapy to induce relaxation in hospitalized patients with incurable cancer: a pilot study. Cancer Nurs.

[75] Cheung DS, Yeung WF, Chau PH (2022). Patient-centred, self-administered acupressure for Chinese advanced cancer patients experiencing fatigue and co-occurring symptoms: a pilot randomised controlled trial. Eur J Cancer Care (Engl).

[76] Yeh CH, Chien LC, Lin WC, Bovbjerg DH, van Londen GJ (2016). Pilot randomized controlled trial of auricular point acupressure to manage symptom clusters of pain, fatigue, and disturbed sleep in breast cancer patients. Cancer Nurs.

[77] Ye Y, Ge J (2021). Clinical application of comfort nursing in elderly patients with advanced lung cancer. Am J Transl Res.

[78] Ma X, Sun S, Zhao Y (2021). Impact of pain care and hospice care on quality of life in patients with advanced gastric cancer. Am J Transl Res.

[79] van der Peet EH, van den Beuken-van Everdingen MH, Patijn J, Schouten HC, van Kleef M, Courtens AM (2009). Randomized clinical trial of an intensive nursing-based pain education program for cancer outpatients suffering from pain. Support Care Cancer.

[80] Shi RC, Meng AF, Zhou WL, Yu XY, Huang XE, Ji AJ, Chen L (2015). Effects of home nursing intervention on the quality of life of patients with nasopharyngeal carcinoma after radiotherapy and chemotherapy. Asian Pac J Cancer Prev.

[81] Jacobsen R, Liubarskiene Z, Møldrup C, Christrup L, Sjøgren P, Samsanaviciene J (2009). Barriers to cancer pain management: a review of empirical research. Medicina (Kaunas).

[82] Oldenmenger WH, Geerling JI, Mostovaya I, Vissers KC, de Graeff A, Reyners AK, van der Linden YM (2018). A systematic review of the effectiveness of patient-based educational interventions to improve cancer-related pain. Cancer Treat Rev.

[83] Koller A, Miaskowski C, De Geest S, Opitz O, Spichiger E (2012). A systematic evaluation of content, structure, and efficacy of interventions to improve patients' self-management of cancer pain. J Pain Symptom Manage.

[84] Plinsinga ML, Singh B, Rose GL (2023). The effect of exercise on pain in people with cancer: a systematic review with meta-analysis. Sports Med.

[85] Cuthbert C, Twomey R, Bansal M (2023). The role of exercise for pain management in adults living with and beyond cancer: a systematic review and meta-analysis. Support Care Cancer.

[86] Ruano A, García-Torres F, Gálvez-Lara M, Moriana JA (2022). Psychological and non-pharmacologic treatments for pain in cancer patients: a systematic review and meta-analysis. J Pain Symptom Manage.

[87] Jane SW, Wilkie DJ, Gallucci BB, Beaton RD (2008). Systematic review of massage intervention for adult patients with cancer: a methodological perspective. Cancer Nurs.

[88] Lopes-Júnior LC, Rosa GS, Pessanha RM, Schuab SI, Nunes KZ, Amorim MH (2020). Efficacy of the complementary therapies in the management of cancer pain in palliative care: a systematic review. Rev Lat Am Enfermagem.

